# Behavioral Assessment of Sensory, Motor, Emotion, and Cognition in Rodent Models of Intracerebral Hemorrhage

**DOI:** 10.3389/fneur.2021.667511

**Published:** 2021-06-17

**Authors:** Xiaoyu Shi, Huiying Bai, Junmin Wang, Jiarui Wang, Leo Huang, Meimei He, Xuejun Zheng, Zitian Duan, Danyang Chen, Jiaxin Zhang, Xuemei Chen, Jian Wang

**Affiliations:** ^1^Department of Human Anatomy, College of Basic Medical Sciences, Zhengzhou University, Zhengzhou, China; ^2^Zhengzhou University Hospital Outpatient Surgery Center, Zhengzhou, China; ^3^Keieger School of Arts and Sciences, The Johns Hopkins University, Baltimore, MD, United States; ^4^Department of Psychology, University of Toronto, Toronto, ON, Canada; ^5^Saint John Paul the Great Catholic High School, Dumfries, VA, United States

**Keywords:** anxiety, behavioral tests, cognition, depression, emotion, intracerebral hemorrhage, motor function, pain

## Abstract

Intracerebral hemorrhage (ICH) is the second most common type of stroke and has one of the highest fatality rates of any disease. There are many clinical signs and symptoms after ICH due to brain cell injury and network disruption resulted from the rupture of a tiny artery and activation of inflammatory cells, such as motor dysfunction, sensory impairment, cognitive impairment, and emotional disturbance, etc. Thus, researchers have established many tests to evaluate behavioral changes in rodent ICH models, in order to achieve a better understanding and thus improvements in the prognosis for the clinical treatment of stroke. This review summarizes existing protocols that have been applied to assess neurologic function outcomes in the rodent ICH models such as pain, motor, cognition, and emotion tests. Pain tests include mechanical, hot, and cold pain tests; motor tests include the following 12 types: neurologic deficit scale test, staircase test, rotarod test, cylinder test, grid walk test, forelimb placing test, wire hanging test, modified neurologic severity score, beam walking test, horizontal ladder test, and adhesive removal test; learning and memory tests include Morris water maze, Y-maze, and novel object recognition test; emotion tests include elevated plus maze, sucrose preference test, tail suspension test, open field test, and forced swim test. This review discusses these assessments by examining their rationale, setup, duration, baseline, procedures as well as comparing their pros and cons, thus guiding researchers to select the most appropriate behavioral tests for preclinical ICH research.

## Introduction

**Figure 1:**
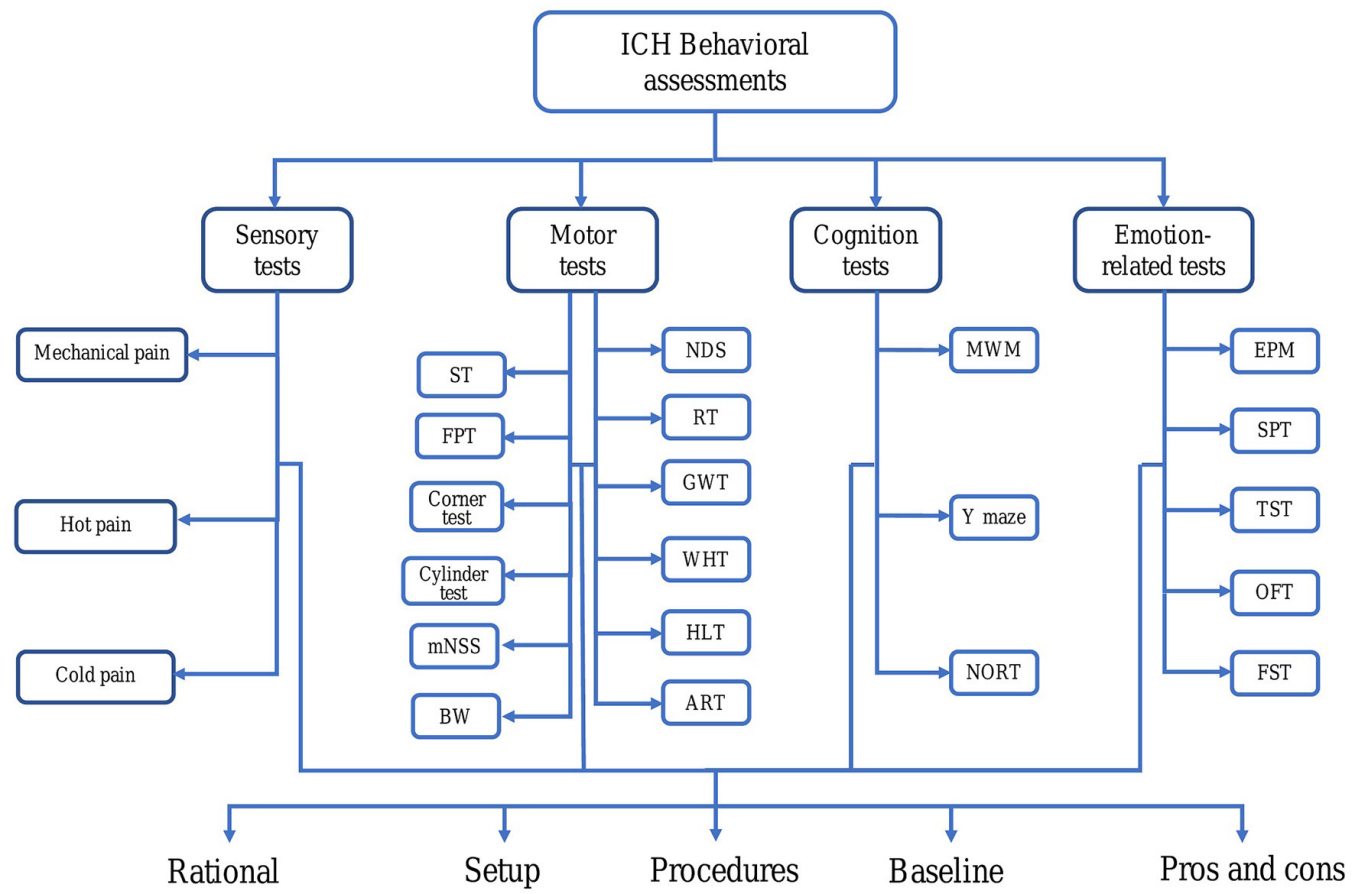
Abnormal behavior after intracerebral hemorrhage (ICH) in rodents. For each behavioral testing, we discussed several aspects, such as rationale, setup, duration, baseline, procedures as well as comparing their pros and cons. ART, Adhesive removal test; BW, Beam walking; EPM, Elevated plus maze; FPT, Forelimb placing test; FST, Forced swim test; GWT, Grid walk test; HL, Horizontal ladder; ICH, Intracerebral hemorrhage; MWM, Morris water maze; mNSS, Modified neurological severity score; NDS, Neurological deficit scale; NORT, Novel object recognition test; OFT, Open field test; RT, Rotarod test; SPT, Sucrose preference test; ST, Staircase test; TST, Tail suspension test; WHT, Wire hanging test.

Intracerebral hemorrhage (ICH) is a type of intracranial hemorrhage which occurs due to sudden rupture of tiny arteries in the brain parenchyma. As the second most common cause of stroke (1, 2), ICH is responsible for 8–15% of all strokes in high-income countries, with the highest rates in Asia (3). An official Chinese report *the National Epidemiological Survey of Stroke* in 2012–2013 showed that stroke is the second most common cause of death within the country; among all causes of stroke mortality, the proportion of ICH was 24% (4). Overall, ICH is a major driver of stroke mortality rates, with a 1-month mortality rate of 30–50% (5), and a one-year rate of 54% (6). Hemorrhage primarily occurs in the basal ganglia, although it can also occur in the thalamus, lobes, brainstem, and cerebellum (7, 8). There are many neurologic manifestations of ICH, depending on the location and the size of the hemorrhage, and the extent of subsequent activation of inflammatory cells (9–11). In general, these manifestations can include motor dysfunction, sensory impairment, and cognitive impairment. Additionally, some ICH patients may have recognition deficits, emotional disturbances, and central pain (12). Thus, to better simulate and evaluate all the aspects of brain cell injury and connecting network dysfunction, a battery of assessments have been established to evaluate behavioral changes in ICH models. Currently, a wide variety of behavior tests exist for the ICH rodent model assessment. However, their documentation in research reports and literature reviews have been sporadic (13). Consequently, a systematic and comprehensive review that summarizes all the applications of various behavioral protocols will allow researchers to be able to more efficiently choose suitable behavioral tests and thus establish effective models to illuminate the pathophysiologic mechanisms and assess the potential translation of ICH treatment. This review summarizes the evaluation methods of sensory, motor, emotion, and cognition tests after the operation of the ICH model in rodent animals (Figure 1), thus allowing experimental researchers to select the appropriate detection methods according to different experimental conditions.

## Common Behavioral Assessments

### Sensory Tests

Sensory tests are used to assess pain sensitivity. Sensory tests generally involve mechanical allodynia, thermal hyperalgesia, and cold hyperalgesia test. The mechanical allodynia test can be performed with dynamic plantar aesthesiometer or von Frey filaments; thermal hyperalgesia is often tested by Hargreaves analgesia meter; and cold hyperalgesia can be tested by acetone test, cold plate test, or cold plantar assay. In these tests, the investigator should be blind to the treatment assignment, and animals should be placed in the test boxes no <30 min in advance to allow for habituation (Table 1). ICH may cause changes in the pain threshold, so pain sensory testing can be used as one of the functional outcome parameters.

**Table 1:** Sensation tests.

**Methods**	**Sub-methods**	**Purpose**	**Timepoint**	**Apparatus**	**Measurement index**	**Range of normal value**
1. Mechanical allodynia	Dynamic Plantar aesthesiometer	Assessment of mechanical allodynia	One day prior to surgery, D1, D3, D5, D7, D10, D14, D21 after surgery (14)	Dynamic Plantar Aesthesiometer, a metal mesh floor, and several Plexiglas chambers	Paw withdrawal threshold (g)	For rats: 35 g (15) For mice: ~0.8 g (16)
	von Frey filaments	Assessment of mechanical allodynia	One day prior to surgery, D1, D3, D5, D7, D10, D14, D21 after surgery (14)	von Frey, a metal mesh floor, and several plexiglass chambers	For rats: paw withdrawal threshold (g) For mice: paw withdrawal percentage: [(number of paw withdrawals/10 trials) × 100%]	For rats: around 15–20 g (16) For mice: around 10% responded to 0.07 g filament stimulation, and 40% to 0.4 g filament stimulation (17)
2. Thermal hyperalgesia		Assessment of thermal hyperalgesia	One day prior to surgery, D1, D3, D5, D7, D10, D14, D21 after surgery (14)	A shelf with a thick glass plate, a radiant heat stimulator, and chambers (16, 18)	The latency of the paw withdrawal response	For rats: ~12 s (15) For mice: ~15 s (18)
3. Cold hyperalgesia	Acetone test	Assessment of cold hyperalgesia	One day prior to surgery, D1, D3, D5, D7, D10, D14, and D21 after surgery	Acetone, 1 ml blunt syringe, and a platform with a mesh floor (19, 20)	A four-point scale	For rats: the total score is below two points (21). For mice: around one point on average (22)
	Cold plate test	Assessment of cold hyperalgesia	One day prior to surgery, D1, D3, D5, D7, D10, D14, and D21 after surgery (23)	An ice-cold metal aluminum platform (23, 24)	The latency of the withdrawal response	For rats: around 25 s (25) For mice: between 15 and 20 s (17)
	Cold plantar assay	Assessment of cold hyperalgesia	One day prior to surgery, D1, D3, D5, D7, D10, D14, and D21 after surgery (18)	A shelf with a glass plate and a 3 ml syringe with the syringe cut the top off (26)	Paw withdrawal latency	For mice: around 10–15 s (26)

### Mechanical Allodynia

Mechanical allodynia is often tested by von Frey filaments or dynamic plantar aesthesiometer, which can well-reflect animals' mechanical pain sensitivity (27, 28). The apparatus needed for this test includes a metal mesh floor and several plexiglass chambers (27). The test is usually carried out 1 day prior to surgery, and 1, 3, 5, 7, 10, 14, and 21 days post-surgery (14). Researchers often utilize the up-and-down method designed by S.R. Chaplan in 1994 (29), but there are also several other methods used in this test. At the beginning of the test, the animals are placed in the chamber on a mesh floor and permitted to freely explore for at least 30 min. Then a dynamic plantar aesthesiometer or von Frey filaments are used to prick the plantar surface of animals' hind paws (16).

For the test using von Frey filaments, ascending forces of filaments are used in sequence. The minimum force that causes the rat to remove its paw is defined as the paw withdrawal threshold. The baseline of sham group rats is around 15–20 g (16, 30). In some experiments, filaments weighing 0.07 or 0.4 g are chosen to test the mice's mechanical pain response (17). Each filament was applied 10 times to the plantar surface (with contact for 3 s), and mechanical allodynia was defined as the percentage of withdrawal responses to the 10 stimulation trials. The paw withdrawal percentage is calculated by the following formula: [(number of paw withdrawals/10 trials) × 100%]. The baseline of the sham group is around 10% with the stimulation of 0.07 g filament, and 40% with the stimulation of 0.4 g filament (17). The paw withdrawal time of the ICH animals should be longer in duration than that of the sham group animals.

As for the dynamic plantar aesthesiometer, the forces will change in ascending, graded forces (2 g/s, and cut-off force is 10 g for mice, 50 g for rats) electrically. The minimum force that causes a paw withdrawal response is recorded. The process is repeated three times for each hind paw, and the mean values are taken as the paw withdraw threshold, respectively (15, 23). The baseline force for normal rats is around 35 g (15), while the normal mice is around 0.8 g (16).

The paw withdrawal threshold declines after ICH. Von Frey test is the most widely used method to test mechanical pain threshold. Its advantage is that it is easy to implement and its own set of standard procedures can facilitate a smooth learning process for beginners. However, a von Frey test takes longer than testing with a dynamic plantar aesthesiometer. Its disadvantage is that both assessments can be biased by factors such as the strength of the hand holding the stab pen, the criteria for judging the sting, the spontaneous movements of animals, etc. (31). Humidity, and probably temperature, may affect elastic modulus, and thus bending force. The filaments should be applied smoothly and avoid miss-hitting the foot or slip. The results can be influenced by the animal's behavior such as grooming or spontaneous movements. In some cases, it is difficult to distinguish whether the animals felt pain or just needed to change their posture. The dynamic filaments may be more easily affected by the postural change (32).

### Thermal Hyperalgesia

The thermal hyperalgesia test requires a shelf with a thick glass plate, a radiant heat stimulator, and plexiglass chambers. The temperature of the plate surface is held constant (14, 16, 18). The test is performed 1 day prior to surgery and on days 1, 3, 5, 7, 10, 14, and 21 post-surgery (14). Before the test, the animals are placed in the chambers for at least 30 min in advance to allow for habituation. The heat stimulator should be focused on the plantar surface of the animals' hind paws under the glass plate (27, 28). The animal will remove its paw when the stimulation reaches the heat threshold. The latency of the withdrawal response (the time between started infrared stimulation to the withdrawal of the hind paw) should be recorded (28). The duration of stimulation should be no longer than 20 s to avoid burn damage (14, 27). The process is repeated three or five times, and the mean values are taken as the threshold values (17, 23, 28). The baseline for normal rats is around 12 s (15) and for mice is around 15 s (18). The latency of the withdrawal response of the ICH animals is shorter than that of the sham group.

The thermal hyperalgesia test is also widely used in pain tests. Its advantage is that when animals remove their hind paws, the device will automatically stop, thus helping to rule out confounding factors such as subjective judgment. Its disadvantage is that other problems might occur because of the free movements of animals as well as their droppings.

### Cold Hyperalgesia

The cold hyperalgesia test includes three typical methods: the acetone test, the cold plate test, and the cold plantar assay (18, 23, 26).

The first one requires a 1 ml blunt syringe and a platform with a mesh floor (19, 20). The animals also need to habituate for at least 30 min in the plexiglass chamber. For the test, acetone is sprinkled on the plantar surface of the animal's hind paw (applied volumes vary across articles) (33). The animal response was observed for 20 s and graded on a 4-point scale (0, no response; 1, quick withdrawal, flick or stamp of the paw; 2, prolonged withdrawal or repeated flicking; and 3, repeated flicking of the paw with licking the plantar surface of the paw). Acetone was applied alternately three times to each hind paw at intervals of 30 s (20, 21). The mean values of the three assessments are taken as the threshold values (21). The baseline values of normal animals are around one point (22). The mean value of ICH animals is higher than that of the sham group.

The cold plate test requires a regular cold metal aluminum platform that can maintain at a constant low temperature (4 ± 1°C) (23, 24). Animals are to be situated on the plate and the latency of withdrawal response is recorded. Withdraw latency is defined as lickings, paw movements, or little leaps (23). A 20 s cutoff for mice (30 s for rats) should be implemented to prevent tissue damage (25). The baseline of sham mice is around 15–20 s, and the baseline of rats is around 25~30 s (17, 25). The latency of the withdrawal response for ICH animals is shorter than that of sham group animals.

There is also a novel behavioral assay for measuring cold sensation in mice, the cold plantar assay. This test requires a shelf with a glass plate a 3 ml syringe with the syringe top cut off, and dry ice powder. Then the dry ice is loaded into the syringe and compacted into an icicle. The icicle is placed on the plantar surface of the mouse's hind paw through the glass plate. The paw withdrawal latency is defined as the period of time between the beginning of contact to when the mouse's foot moves away. The baseline latency for normal mice is around 10–15 s. This duration is shorter in ICH animals. The cold plantar assay can complement currently used assays and accurately measure the cold response threshold (26).

Cold hyperalgesia tests are widely used in ICH models. Although the acetone test is easy to administer, it has poor precision since the capacity of most syringes is 1 ml whereas the experimental dosage of acetone needed is usually only 0.025 ml. The rate at which acetone is ejected from the syringe can also adversely affect the tests. In addition, acetone may have adverse health effects for the animals and researchers. In contrast, the cold plate test is more precise and objective, but more expensive. The advantage of the cold plantar assay is that it is easy to utilize and cheap, but it hasn't been widely used as the other two tests.

### Motor Tests

Presently, motor tests commonly used for ICH detection in basic research generally include the following 12 types: the neurologic deficit scale, corner test, staircase test, rotarod test, cylinder test, grid walk test, forelimb placing test, wire hanging test, modified neurologic severity score, beam walking test, horizontal ladder test, and adhesive removal test (Table 2).

**Table 2:** Motor tests.

**Methods**	**Purpose**	**Timepoint**	**Apparatus**	**Measurement index**	**Range of normal value**
1. Neurologic deficit scale	Use in motor function tests	D1, D3, D7, D14, and D21 post-ICH (34, 35)	None required	Each aspect is graded from 0 to 4 points, and the total score ranges from 0 to 24 points	0 point in both mice and rats (34, 35)
2. Corner test	Assessing integrated sensorimotor function	One day prior to surgery, D1, D3, D7, D14, D28 and D21 post-ICH (34, 35)	Two cardboard pieces forming a corner with a 30° angle (36)	The percentage of corner turn scores	Around 50% in both mice and rats (36–39)
3. Staircase test	Measuring spontaneous forelimb usage, walking, and skilled reaching ability	D6, D28, D29, D30, D31, and D32 post-ICH (40)	A plexiglass box with several ladders (40)	The number of pellets remaining in each well on the two sides	Above nine in both rats and mice (41, 42)
4. Rotarod test	Conduction of a double-blind assessment of behavioral function	D1, D3, D7, D14, and D21 post-ICH (43)	An accelerating rotarod (43)	The average retention time of staying in the rotarod	For rats: ~150 s (44) For mice: around 250~300 s (45)
5. Cylinder test	Measurement of brain function and assess spontaneous forelimb use in rodents	D1, D3, D7, and D14 post-injury (46)	Transparent acrylic glass cylinder with a diameter of 7 to 10 cm (46)	Results analyzed with the following formula: (contralateral forelimb movement—ipsilateral forelimb movement)/(contralateral forelimb movement + ipsilateral forelimb movement + both movement)	Around 0 in normal animals (47)
6. Grid walk test	Measurement of sensorimotor coordination in mice	D1, D3, D7, and D14 post-injury (48)	An overhead grid which connects two tall walls (15)	For rats: the numbers of foot faults For mice: a foot fault index [(Contralateral faults – Ipsilateral faults)/total steps] is calculated	For rats: ~20 times (49, 50) For mice: <5% (51)
7. Forelimb placing test	Assessment of ICH - induced neurological deficits	D1, D3, D12, and D28 post-ICH (52, 53)	None	This forelimb placement experiment was quantified as the percentage of successful responses in 10 trials	Nearly 100% in both rats and mice (54, 55)
8. Wire hanging test	Evaluation of locomotor abnormalities and behavioral deficits in models of striatal, intra-ventricular, and cortical ICH	D1, D3, D7, D14, and D21 post-ICH (56)	A temperature-controlled and humidity-controlled room, an iron wire (1 mm in diameter, 55 cm long, 50 cm above the ground) (57)	The time that each animal remained on the wire is recorded, and the average time is calculated	Around 35 s in mice (58)
9. Beam walking test	Measurement of balance and asymmetrical coordination	D1, D3, D7, D11, D14, D21, and D28 post-ICH (59)	A wooden beam usually 50 cm above the ground for mice, 1 m above the ground for rats (59–62)	Seven point scale	Seven point (63)
10. Horizontal ladder test	Evaluation of walking ability	D7, D14, D21, D28 post-ICH (64–66).	A horizontal ladder 30 cm above the ground, and made up of two clear side walls (1 m long and 19 cm high) and several metal rungs (3 mm in diameter) (67, 68).	Limb error rate = error steps/total steps × 100%	Close to 0 (68)
11. Adhesive removal test	Evaluation of sensorimotor neurologic deficits of both forepaws	D1, D3, D7, D14, D21 and D28 post-ICH (69, 70)	Small adhesive tape pieces (around 4 mm for mice, 6 mm for rats) (64, 70–72)	The latency of removing the tape is recorded	Within 10 s (73)

### Neurologic Deficit Scale

The 24-point neurological deficit scale is often used in the motor function tests. It is the most convenient assessment and doesn't require any apparatus. The animals are tested on days 1, 3, 7, 14, and 21 post-ICH (34, 35). For mice, the researcher should observe 6 aspects, including body symmetry, gait, climbing, circling behavior, front limb symmetry, and compulsory circling. Each aspect is graded from 0 to 4, with the maximum score being 24 points (74). As for rats, the researcher should observe spontaneous ipsilateral circling, hind limb retraction, bilateral forepaw grasp, beam walking ability, forelimb flexion. The first four assessments should be graded from 0 to 3, the last one should be graded from 0 to 2. The maximum score is 14. The detailed scoring protocol has been described previously (75). The baseline for both normal and ICH animals is 0 points. The advantage of the neurological deficit scale scoring system lies in its simplicity of procedure as well as equipment and having a very exact criterion.However, it can be influenced by the animals' autonomous activities and the subjective judgments of the researchers (76).

### Corner Test

The corner test is a method used to assess sensory-motor function, which has been proven to be a reliable method for identifying as well as quantifying sensory and postural asymmetry (74, 77). It provides a simple method for the detection of contralateral deficit and ipsilateral steering deviation.

The test was applied to the unilateral nigral striatum injury in rats (78) and to the focal cerebral ischemia in mice (36). The device is tightly attached to two adjacent plastic plates to form a narrow lane of 30 degrees, leaving a small opening to approach, and then the animals are put between the plastic plates facing the corner. When animals approach the corner, both sides of the whiskers are stimulated at the same time, causing the animal to rotate 180 degrees. There is no marked preference for turning direction in healthy animals whereas unilaterally brain-damaged animals may display a consistent preference for the same side. The baseline for normal animals is around 50% (36–39).

The corner test is simple, impartial, and relatively sensitive (36). In addition to identifying sensorimotor disorders, corner testing has been shown to be an objective assessment of long-term functional outcomes in rats and mice (up to 90 days) after stroke (41, 42, 79). There is an advantage that the corner test may be more sensitive than other symmetrical tests because it reflects multiple asymmetries, including forelimb, hind limb, posture, and steering bias (37). However, it is not sensitive for severely injured animals and repeated tests (76).

### Staircase Test

The staircase test is mainly used to measure and compare the flexibility, motor coordination, and autonomy ability of mice before and after forelimb dysfunction caused by ICH (40). Its main advantage is that it can independently evaluate the ability of mice to skillfully use their limbs after injury.

This test was originally designed to assess the independent use of forelimbs in rats (40) and was later used to assess skilled reaching (80). By observing rats' behavior when they reach for food pellets, bilateral measurements of animal forelimb stretch, mastery of skills, and lateral deviation can be quantified.

The apparatus including a plexiglass box (19 cm wide × 27 cm long × 25 cm high) with an elevated platform in it, and seven stairs descending on each side of the platform (40). Food pellets (45 mg of each pellet) were placed on the stairs (three pellets for each stair). The rat rests on the platform and the food pellets on one side can only be reached by the rat's paw on the same side. The device is designed to encourage animals to get food through confined spaces (40). The rats are generally food-deprived 3 days before the assessment, and trained for 2–3 weeks before this task (5 days per week, two 15-min trials a day with a 4–5 h duration) (64, 81). Normal animals can usually collect pellets quickly (77). The number of pellets collected is recorded. The range of normal values is above nine pellets on each side in both mice and rats (64, 80). This test has been proven to be sensitive to persistent defects in the detection of ischemic brain injury (40, 81). Potential drawbacks of this test are that detailed behavior cannot always be well-quantified (40) and that long-term pre-training is needed (77). The video of detailed procedures for rats is available in reference (82).

### Rotarod Test

The rotarod test is used to assess motor coordination and balance (83, 84). This experiment includes two parts: 3 days of training before surgery and formal tests on days 1, 2, 3, 5, 7, and 14 after surgery. Before starting the rotarod test, the mice are trained for 15 min at a set rotational speed (15 RPM), followed by three trials accelerated from 4 to 40 RPM in 5 min (43, 85). The average baseline latency of the three training days before the operation is obtained (43, 69). On the test day, three trials are run on each animal, and the average retention time of three trials is computed (43). There should be a 15-min rest interval between each test (46). Before surgery, the baseline of rats is around 150 s (86), while the mice are around 250~300 s (45, 87). Animals with hemorrhagic damage tend to fall faster than normal ones (88). The video of detailed procedures for rats is available in reference (89). The video of detailed procedures for mice is available in reference (90). Rotarod test is sensitive and straight forward, but is unable to evaluate the acquisition of motor skill learning (91).

### Cylinder Test

The cylinder test can not only be used as an assay of brain function but can also evaluate rodent's spontaneous forelimb use, which is the main advantage of this method. The device consists of a transparent acrylic glass cylinder with a diameter of 7–10 cm. Two mirrors are placed behind the cylinder to observe the mice from three different angles at the same time. In each trial, the instances of placement of left and right, right, or left forelimbs on the wall are recorded. The average percentage of baseline use of the damaged forelimb is reported to have been tested for two 5-min trials, up to 20 times for each. The animals are evaluated twice prior to surgery for baseline and then at days 1, 3, 7, and 14 days post-injury (47). The results are analyzed with the following formula: (contralateral forelimb movement - ipsilateral forelimb movement)/(contralateral forelimb movement + ipsitralateral forelimb movement + both movement). The baseline for normal animals is 0 (47). The use of contralateral claws is reduced in animals with brain damage. The video of detailed procedures for mice can be seen in reference (92). The video of detailed procedures for rats can be seen in reference (82). The cylinder test is low-costing and easy to perform. It is sensitive to assess chronic deficits (93), but is not under-utilized to assess forelimb deficits (92).

### Grid Walk Test

The grid walk test (GWT) is a sensitive measure of sensory-motor coordination (94). The apparatus for this test is consists of an overhead grid that connects two tall walls (15). The opening of the grid for rats is around 2–3 cm (95). The animals should be evaluated at days 1,3,7, and 14 post-injury (48). Normal mice will precisely grasp the wireframe to balance themselves when placed on mesh while the hemorrhagic mice's paws may slip through the open grid, this is defined as a “foot fault” (94). For the test, the animals are placed on the mesh for 3 min while the number of “foot faults” for each limb and their total steps are recorded. The average value of the foot fault test in normal rats is around 20 times (49, 50). A foot fault index [(contralateral faults—ipsilateral faults)/total steps] is calculated. 0 represents no asymmetry; a positive score indicates increased contralateral foot faults and implies impaired contralateral motor function. Since the injury of the unilateral brain will cause contralateral neurologic deficits (77), animals would show increased contralateral foot faults after unilateral ICH. The normal occurrence for slips is < ±5% in the sham mice (51). You can watch the video of detailed procedures for mice as provided by reference (96). The video of detailed procedures for rats is available from reference (97).

### Forelimb Placing Test

The forelimb placing test (FPT) was scored using the vibrissae-elicited forelimb placing test (98). Mice have been known to respond to vibrissae stimulation with foreleg movement (59). Thus, this can be used to assess ICH-induced neurological deficits. Prior to the test, the animals were gently moved up and down to promote muscle relaxation and to eliminate any struggle response (37, 99). Next, they are placed on the edge of the table. When the mice's vibrissae touch the table, the healthy animals will quickly place their ipsilateral forelegs on the table. This forelimb placement experiment can be quantified as the percentage of successful responses in 10 trials. It usually takes 5 min to complete the test. In general, this test was performed on days 1, 3, 12, and 28 after ICH (52, 53). Animals with unilateral brain damage have been found to respond less on the contralateral side while healthy rats will generally have a higher success rate in this task (98). The baseline performance for sham group animals is nearly 100% (54, 55). Overall, the advantage of this test is that it is quick and easy to perform. It can detect mild neurologic impairments (77). The examiners should practice in advance to avoid abrupt moving. The integrity of the mouse whiskers is a prerequisite for this test (77).

### Wire Hanging Test

The wire-hanging test (WHT) is useful for evaluating locomotor abnormalities (100). It is applied to evaluate grip strength, balance, and endurance in mice on days 1, 3, 7, 14, and 21 post-ICH (56). An iron wire (1 mm in diameter, 55 cm long) is stretched horizontally between two posts, 50 cm above the ground. Mice are placed on the wire and have to use their forelimbs to suspend their body weight. The hind limbs are gently covered with adhesive tape to prevent them from using all four paws. A pillow is placed beneath the mice to prevent falling injuries. The time that each animal remained on the wire is recorded (57). The baseline of the sham group is around 30–40 s (101). The result is represented as the average of three trials per animal (102). Compared to normal mice, gripping and forelimb strength are significantly impaired in ICH mice at all of the time points, and falling latency in the wire-hanging test is shorter than normal or sham animals on days 1, 3, and 7 post-ICH (56). WHT is useful to measure coordination and endurance. The limitation of this test is that it's unsuitable for rats. For their heavier weight means that it is painful for them to support their body weights on a wire and the test has a higher chance of causing fall injuries. Results might be in inconsistent because of the moving of the hind limbs (103).

### Modified Neurologic Severity Score (mNSS)

The modified neurologic severity score (mNSS) contains sensory tests, motor tests, reflex tests, and beam balance tests (104) and is used to assess neurologic deficits and the grade of neurologic damage on the aspects of motor, ground walking, sensory, coordination of movements, reflex, and abnormal movements (105) (Table 3). The tests are performed on days 1, 3, 7, 14, and 30 after ICH by the testers who are blind to the treatment groups using either rats or mice (106). For both mice and rats, the tester should observe 4 aspects, including abnormal movements or absence of reflex, beam balance test, sensory function, and locomotor function. The first, third, and fourth aspects are all graded from 0 to 1 point and the second aspect is graded from 0 to 6 points. The baseline for the normal animals is 0 points. Each animal should be tested twice and the average score is calculated after the test (105). The highest score is 18. The higher the score, the more severe the injury is (104).

**Table 3:** The modified neurologic severity score (mNSS).

**Methods**	**Purpose**	**Timepoint**	**Apparatus**	**Measurement index**	**Range of normal value**
1. Abnormal movements or absence of reflex	Assessment of neurologic impairment on reflex and abnormal movements	One day prior to surgery, D1, D3, D7, D14, D30 after surgery (106)	None required	Corneal reflex, pinna reflex, startle reflex, and dystonia or convulsion	0 point in both mice and rats (105)
2. Beam balance test	Assessment of neurologic impairment on coordination of movements	One day prior to surgery, D1, D3, D7, D14, D30 after surgery (106)	Wooden cylindrical bar (107)	The time that stays on the balance beam	0 point in both mice and rats (105)
3. Sensory function	Assessment of neurologic impairment on sensory function	One day prior to surgery, D1, D3, D7, D14, D30 after surgery (106)	A table	Contractile reaction	0 point in both mice and rats (105)
4. Locomotor function	Assessment of neurologic impairment on locomotor function	One day prior to surgery, D1, D3, D7, D14, D30 after surgery (106)	None required	The state of the motor function	0 point in both mice and rats (105)

### Beam Walking

Beam walking is always carried out to measure balance and asymmetrical coordination (108). It can be performed at 1, 3, 7, 11, 14, 21, and 28 days post-ICH (59). The wooden beam sets up at 50 cm above the ground for mice and at 1 m above the ground for rats (59–62). Animals should be trained to cross the beam before surgery (107). The animals are graded on a seven-point scale on the performance when they cross the beam. Each testing session consists of three trials to get an average value (63, 65, 109). This test can be used to evaluate the balance and the locomotor activity of the rodents. The disadvantage of this test is that the narrower the balance beam is, the more times the test animal misses, which leads to the lower reliability of the results.

### Horizontal Ladder Test

The horizontal ladder test (HLT) is used to evaluate walking ability (110, 111). Rats are mainly used in this test. The horizontal ladder is 30 cm above the ground and is made up of two clear side walls (1 m long and 19 cm high) and several metal rungs (3 mm diameter). The distance between two adjacent rungs is changeable (1–5 cm accordingly) (67, 68). A home cage is placed at the end of the ladder to encourage the animals' moving (112). Rats should be trained for 3 days before surgery and tested at 7, 14, 21, and 28 days post-surgery, and three times per day to get an average value (64–66). Every slight paw slip, deep paw slip, and complete misses are scored as an error (112, 113). The total number of steps and the number of errors of each limb is counted (113). Each limb error rate is calculated as error steps/total steps × 100%. The baseline of normal animals is closed to 0 (68). HLT is sensitive enough to evaluate the relationship between motor impairment and injury volume in ICH (67). It is useful in assessing chronic deficits and can be used repeatedly since the rungs are changeable (112).

### Adhesive Removal Test

The adhesive removal test (ART) is used to assess the sensorimotor neurologic deficits of both forepaws (69, 114). It is assessed on days 1, 3, 7, 14, 21, and 28 post surgery, and needs 3 days' training prior to surgery (69, 70). The equipment needed for this test is very simple, the adhesive tape and a scissor. Using the scissor to cut the tape into small circular or square pieces, the diameter or length is around 4 mm for mice and 6 mm for rats (64, 70, 71). Gently apply the tape to each forepaw in a random order (60, 115). Be sure to keep equal pressure between each trial and each animal (60). The latency of removing the tape is recorded from the time the animal notices the tape until the tape is removed. The animal's forepaw is tested three times to calculate the average value. An interval of 5 min between each trial is necessary (73, 115). The cutoff time is around 120–180 s (60, 64, 115). The animal should acclimatize the experimental cage for 2 min in advance (69, 116). A 3-day-training is required to ensure the animal adapts to the condition of being put on the tape, and to learn to tear the tape off within 10s (73). ART can be used to evaluate primarily the sensory deficits and the asymmetrical biases (79). Special equipment is not needed. The size and stickiness of the tape should be the same for data reproducibility (93).

### Cognition Tests

A variety of mazes are widely used in testing animals' cognitive ability. In basic research of ICH rodent animal models, the Morris water maze, Y-maze, and novel object recognition tests are the most frequently used methods (Table 4).

**Table 4:** Cognition tests.

**Methods**	**Purpose**	**Timepoint**	**Apparatus**	**Preparation**	**Measurement index**	**Range of normal value**
1. Morris water maze	Evaluation of spatial learning and memory ability	D8-15 post-ICH (117, 118)	A metal pool (110 cm in diameter) filled within 15 cm of the upper edge, a platform (11 cm in diameter) for the animals to escape to the changing position of each block (maximum = 60 sec/test)(119)	Mice are trained at intervals of 20–30 min for a total of four times during each training day (117, 118)	Escape latency, percentage time spent in the target quadrant, and platform crossing times.	For rats: escape latency: ~15 s in the testing day (88)For mice: escape latency: ~45 sTarget crossings: 12 times (119)
2. Y-maze test	Testing of spatial memory	D30 post-ICH (120)	Consists of three arms (40 cm × 15 cm × 35 cm for rats, 30 cm × 10 cm × 17 cm for mice) diverging at a 120° from the central point, and the entrance of each arm is closed with a baffle (75, 121)	Placement of animals inside the arm for free exploration of the opened two arms for 5 min (120, 122).	The percentage of novel arm entries.	Around 35% in mice and rats (75, 108)
3. Novel object recognition test	Testing of non-spatial memory	D21 post-ICH (57)	Three objects numbered A, B, and C respectively, and an open-field arena (30 cm × 25 cm × 20 cm) (57)	Habituation to the environment for 5 min 1 day before the test (57, 123)	The discrimination index (exploring object C/exploring of both objects)	Around 70% in mice and rats (57, 123)

### Morris Water Maze

The Morris water maze (MWM) is usually used to evaluate spatial learning and memory ability after ICH (117). The required device consists of a metal pool (120 cm in diameter; 55 cm in height) which is divided into four quadrants with a platform (10 cm in diameter, 21 cm in height) in one quadrant for the animals to escape. The pool is filled with water 2 cm above the platform. The water temperature is maintained at 26 ± 1°C (58, 118). Noldus EthoVision tracking software is to record the delay, frequencies, and swimming speed of the mice before the discovery of the platform (119). The experiment includes two parts: 5 days of training and a sixth test day. The mice are trained at intervals of 20–30 min for a total of four times during each training day. During training, mice should be placed gently on the instrument facing the wall. On average, the mice found the platform within 90 s and stayed on the platform for 15 s (118, 124). If the platform could not be found within 90 s, the mice are gently guided to the platform for 15 s, and the latency is recorded as 90 s. After each test, mice are wiped with a towel and placed into the heating cage. On the sixth day, the platform is removed, and the animals are tested as usual. The results are the time spent in the incubation period and the target quadrant of the platform (117, 118, 125). In training experiments, shorter latency in reaching the platform can be correlated with better spatial learning and memory ability. Initially, ICH mice took significantly longer than control mice. The escape latency of sham group rats is around 15 s (88). For the sham group mice, the escape latency is around 45s, and target crossings required ~12 s (119). However, this latency should significantly be reduced over the next few days, suggesting that spatial memory is established (126).

MWM is the most widely used method for testing memory (127). Olfactory trails or cues are eliminated in this test (128). The experimental data can accurately assess the animal's sense of spatial position and direction, especially in terms of spatial positioning. However, this method has some shortfalls. Swimming is an acute stressful stimulus for the animals, so neuroendocrine effects may impact experimental results (128). Additionally, the water temperature has a significant impact on animal activities, so it is essential to keep the water at a comfortable temperature. Finally, after the experiment, the experimental animals should be dried immediately to prevent sickness. The video of detailed procedures for mice can be seen in reference (129). The video of detailed procedures for rats can be seen in reference (130).

### Y-Maze Test

The Y-maze is also often used to test spatial memory. The device of Y-maze consists of three arms [40 cm long × 15 cm wide × 35 cm high for rats, 30 cm long × 10 cm wide × 17 cm high for mice)], diverging at 120° from the central point (121). The entrance of each arm is closed with a baffle. The test is performed 30 days after the operation (120). During the test, the baffles of two random arms are opened. One of the two is chosen to be the Start arm, and the remaining arm with baffle still on is designated the novel arm (122). The animals are put in the start arm and allowed to explore the opened two arms freely for 5 min. Then they are put back in the home cage to rest. After 2 h, all three baffles are removed and the animal is allowed to explore freely for 5 min. The durations of explorations in three arms and the duration in each arm are recorded (117). The percentage of novel arm entries is calculated around 35% (108, 121). There is another calculation method. First, label the three arms of the maze A, B, and C. Then start recording. Next, examine the recorded number of all arm entries and alternations. Finally, calculate the percent (%) alternation with the following formula:

% Alternation = (Number of Alternations/[Total number of arm entries − 2]) × 100 (131).

The advantage of the Y-maze test is that it is easy to perform and the apparatus itself is simple and convenient. The sensitivity and reproducibility of the test need to be characterized in the ICH models (76). The video of detailed procedures for mice can be seen in reference (132). The video of detailed procedures for rats can be seen in reference (133).

### Novel Object Recognition Test

The novel object recognition test (NORT) assesses the animals' memory capability. It requires three objects, numbered A, B, and C, respectively, and an open-field arena (47 cm × 26 cm × 20 cm) that can hold the objects and mouse (56). Object A is the same as object B (green cubes, 4 cm × 4 cm × 3 cm), and they both look different from object C (white ball, 5 cm in diameter) which is the novel object (56). The open-field arena for rats is larger (60 cm × 60 cm × 50 cm) (134). The test includes three periods: habituation on day 1, training on day 2, and testing 1 h after the training. First, the mouse is placed in the empty arena and allowed to explore for 10 min on day 1. On the next day, objects A and B are placed in the open-field arena, and the mouse is put between these two objects, where it is permitted to explore freely for 5 min. Then, the mouse is taken out and placed back in its cage for a rest. The arena should be cleaned with alcohol to eliminate other scents at this time. After 1 h, object B is replaced by object C, and the mouse is returned to the arena for 5 min. The durations of exploration for object A and object C are recorded. The movement of the mouse is recorded by a camera and analyzed later. Sniffing or touching with the nose and/or forepaws within 2 cm around the objects is defined as an exploration event. Sitting or leaning on the objects is not considered to be exploratory behavior. The discrimination index is calculated as the time spent exploring object C divided by the time spent exploring both objects. The baseline of the discrimination index in normal mice or rats is around 70% (57, 123).

NORT is simple to perform and can be completed in a short time (135). However, experimental data is painstaking to obtain since video analysis takes considerable time and the animals' exploratory behavior may be hard to categorize at times (57). Choosing appropriate objects is difficult because the size, shape, material, and height, etc. may affect animals' preference (76). The video of detailed procedures for mice can be seen in reference (136). The video of detailed procedures for rats can be seen in reference (137).

### Emotion-Related Tests

Emotion tests are currently commonly used in basic neurologic research but seldomly used in ICH research. The mood tests are mainly divided into anxiety and depression tests. These texts include the elevated plus-maze, sucrose preference test, tail suspension test, open field test, forced swim test, and so on (Table 5).

**Table 5:** Emotion-related tests.

**Methods**	**Purpose**	**Timepoint**	**Apparatus**	**Measurement index**	**Range of normal value**
1. Elevated Plus Maze	Testing for anxiety	D30 post-surgery (75).	Consists of two arms and looks like a cross, one of the arms has walls around, called an enclosed arm, while the other without walls is the open arm. The maze for the rat is 50 cm × 10 cm × 50 cm [86]. The maze for the mice is 45 cm × 11 cm × 22 cm, and 80 cm above the ground (138)	The percentage of time spent in the open arm and the entries into the open arm	For rats: the percentage of time spent in the open arm: around 30% (139) For mice: the number of entries into the open arm: around 3 times (140).
2. Sucrose preference test	Testing for anhedonia and depression	D18-21 post-surgery (141)	Consists of two bottles, one of which is used to hold 1% sugar solution and the other holds pure water (141)	The sugar solution preference is calculated by following formula: the sugar solution consumption (g)/[pure water consumption (g) + sugar solution consumption (g)]	For rats: ~70% (142) For mice: around 90% (56)
3. Tail suspension test	Testing for depression	D21 post-surgery (57)	A hanging box (55 cm × 60 cm × 11.5 cm), Polycarbonate tube (4 cm in length, outside diameter 1.6 cm, inner diameter 1.3 cm), and packaging tape (57)	The duration of stationary time	150–300 s in mice (143)
4. Open field test	Testing for anxiety-like emotion in post-stroke pain models	D30 post-surgery (75)	An open-field box (100 cm × 100 cm × 100 cm), the bottom of which is subdivided into 16 equal squares, and a computerized tracking system (144, 145)	The duration in the outer and inner zone	For rats: ~50 s in the inner zone (145). For mice: the duration in the outer zone is around 350 s, and in the inner zone is around 200 s (144)
5. Forced swim test	Analysis of depressive-like behavior	D22 post-surgery (146)	A container (50 cm high and 20 cm in diameter for rats, 20 cm high and 22 cm in diameter for mice) filled with water (146, 147)	The duration of immobility, climbing time and swimming time	For rats: the mean immobility time of 125–150 s, a mean struggling time of 75–100 s, and a mean swimming time of 200–225 s (147) For mice: a mean immobility duration of 120–140 s, a mean climbing time of 20–40 s, and a mean swimming time of 70–90 s (146)

### Elevated Plus Maze

The elevated plus maze (EPM) is generally applied to test anxiety (75). The test apparatus is composed of two arms and looks like a cross. One of the arms surrounding walls and is called the enclosed arm, while the arm without walls is called the open arm. The maze for the rats is 50 cm × 10 cm × 50 cm (75). The maze for the mice is 45 cm × 11 cm × 22 cm and located 80 cm above the ground (138).

The test relies on the rodent's exploratory nature. When placed in the middle of an EPM, their nature compels them to examine the open arm. However, rodents' fear of heights would discourage exploration, thus inducing anxiety. The test is performed 30 days post-ICH surgery (75). Two hours before the test, the mice are put into the testing room to adapt to the environment. At the start of the test, the animal is placed at the intersection of two arms with its head facing the open arm. The animal is allowed to explore the maze freely for 5 min (138, 148). The time spent in the open arm and the number of entries into the open arm is recorded (149). For rats, the baseline for entries into the open arm is around three times, and the percentage duration of time spent in the open arm is around 30% of the total testing time (139). For mice, the duration of time spent in the open arm is around 20 s, with ~3 entries (140).

The advantage of the EPM test is that it is easy to conduct and record, and does not need pre-training (150). However, sometimes animals can fall from the open arms and get hurt, and some animals may be reluctant to move onto the arms after surgery. The video of detailed procedures for mice is available from reference (151). The video of detailed procedures for rats is available from reference (152).

### Sucrose Preference Test

The sucrose preference test (SPT) is used to test anhedonia and depression (141). The device includes two bottles, one of which contains 1% sugar solution and the other pure water (141). The test is performed at 18–21 days post-surgery (141). At the beginning of the test, two bottles of water of equal weight are placed in the cage. At the end of the test, the bottles are weighed and water consumption is calculated. Then the sugar solution preference is calculated by the following formula: the sugar solution consumption (g)/[pure water consumption (g) + sugar solution consumption (g)] (153). The baseline of normal rats and mice is around 70% (142) and 90% (56), respectively. We conclude from the references that compared with the control group, the sugar solution preference degree of the depression group is lower (154).

SPT is easy to perform and causes no harm to the animals, It is the best method for evaluating anhedonia in mice (155). However, it lacks standardized apparatus (156). Water loss when taking out and putting in the cage is not easy to control, which will affect the experimental results. The experimental results are closely related to the feeding state of animals and can be affected by environmental inconsistencies (156). Researchers have to control the animals' diet before the test. The video of detailed procedures for mice can be seen in reference (157). The video of detailed procedures for rats is available from reference (158).

### Tail Suspension Test

The tail suspension test (TST) is applied to test depression (159). The apparatus consists of a hanging box (55 cm × 60 cm × 11.5 cm), polycarbonate tube (4 cm in length, outside diameter 1.6 cm, inner diameter 1.3 cm), and packaging tape. The test is performed on day 21 post-surgery (57). To prepare for the test, a 17 cm tape with a marking at 2 cm is stuck to the animals' tails (2–3 mm of the end of the tail should be left outside the tape). The free end of the tape is hung on the hanging rod. The upside-down position will cause the animals to struggle. When they come to realize that struggling does not change their situation, they will cease to move. The mice are suspended for 7 min, and the stationary time in the last 6 min is recorded (143). After the test, the mice should be put back into their cages, and the researcher should gently pull off the tape from the tail. It is concluded from the references that the immobility time in 6 min of the control group is 150–300 s.

TST is easy to perform (160). The limitation of the test is that it is not suitable for rats because they are heavier. Thus, it is painful for them to rely on their tails to support their bodies' weight (tail fracture is a possibility).

### Open Field Test

The open-field test (OFT) is often used for locomotor activity and anxiety-like emotion in ICH models (13, 161). The apparatus consists of an open-field box (50 cm × 50 cm × 38 cm), the bottom of which is subdivided into 25 equal squares (nine squares in the center called the inter-zone, surrounded by 16 outer zones), and a computerized tracking system (144). Like the EPM test, the OFT utilizes the exploratory nature of rodent animals. Normal animals will spend time exploring the inner zone, while ICH animals are more likely to stay in the outer zone to feel more at ease. The test is performed on day 30 after surgery (75). On the testing day, the animals are put into the testing room for 2 h in advance to adapt to the testing environment. When the tests begin, the animals are put into the box for 10 min (145). The duration of time spent in the outer and inner zones is recorded (145, 161). The baseline duration spent by normal mice in the outer zone is around 350 s, and the duration spent in the inner zone is around 200 s (144).

OPT is widely applied in rodent behavioral research, but it has limitations because its outcome can be easily affected by many factors, such as time, lighting conditions, and room temperature (76, 144). The video of detailed procedures for mice is available from reference (144).

### Forced Swim Test

The forced swim test (FST) is a behavioral test for rodents first described in 1978 by Porsolt et al. (162). This test was developed as a model for predicting the clinical efficacy of antidepressant drugs and is now also widely used to analyze depressive-like behavior (57, 146).

In this test, an animal is placed in a container (50 cm high and 20 cm in diameter for rats, 20 cm high, and 22 cm in diameter for mice) filled with water. The test for rats consists of two sessions 24 h apart. The first session is the pre-test stage (15 min) and the second session is the test stage (5 min). The test for mice consists of one session 6 min long, divided into two sessions; the pre-test (the first 2 min) serves as a habituation period for the test (the last 4 min) (146). Immobility, swimming, and climbing behaviors are recorded (163). References indicate that normal mice have a mean immobility time of 120–140 s, a mean climbing time of 20–40 s, and a mean swimming time of 70–90 s (146). The baseline for normal rats is 125–150 s of immobility time, 75–100 s of climbing time, and 200–225 s of swimming time (147). It has been proven that animals exhibit increased immobility time in the FST after depression and that various antidepressants are able to reduce immobility time by increasing the swimming and/or climbing time (147, 164).

FST is low-costing, fast, and reliable. It is widely used to screen anti-depressants (160). However, it lacks construct validity and specificity (93, 165). Experimental animals will suffer from behavioral desperation when they are forced to swim. Additionally, one crucial limitation of the FST is that re-testing will lead to inaccurate experimental results, so animals can only be tested once in their lifetime. Lastly, similar to other tests, animal behavior in the FST is also influenced by biological factors including preconditioning before the FST, schedule, routes of treatment, dosage, and type of drugs, experimental design and, laboratory environmental factors (166). Detailed video for the mouse (167) and rat (146) procedures are available.

Finally, due to the great variety of animal behavior tests, more effort should be made to ensure the consistency of experimental conditions, and more attention should be paid to the following aspects to reduce the error of experimental results. (1) The test environment should be quiet and appropriate. (2) The animals should be given 2–3 h to habituate to the experimental environment before the test. (3) Animals should have rested for sufficient intervals to eliminate influence from the previous experiment. (4) The experimental equipment is wiped with alcohol before testing to eliminate odor. (5) External stimuli such as lighting, water temperature, water quality, and movement of the experimenter may all influence the animal's behavior. (6) Animals soaked in water should be dried at the end of the experiment to avoid sickness. (7) Double-blind should be taken as far as possible to reduce the influence of subjective factors. (8) Animals with significant limb use bias during pre-training should be excluded from the test.

## Concluding Remarks

Acute ICH increases with age and can occur in various brain locations. Subsequent brain damage and network disruption can lead to location-specific clinical signs and symptoms. Therefore, a wide range of behavioral tests should be utilized to assess relevant functional impairment. For example, when the striatum is injured, the sensorimotor function should be assessed, and when the thalamus is injured, the pain and emotional responses in addition to the motor function should be assessed.

Currently, the collagenase-induced and the whole blood animal models are the two best simulations of clinical ICH. They both generate hematoma within the brain parenchyma with distinct pathophysiology. Based on our knowledge, there are no studies focusing on the differences between these two preclinical models of ICH with regard to behavioral aspects. Our unpublished data indicate that, when comparing with the whole blood ICH model, the collagenase-induced ICH model shows greater blood-brain barrier breakdown and more severe neurologic deficits. Comparing with the collagenase-induced striatal ICH model, the collagenase-induced cortical ICH model shows transient and mild neurologic deficits and greater cognitive and emotional impairment (57). It has been suggested that both the collagenase-induced and the whole blood model should be tested in preclinical ICH drug efficacy studies.

There is currently no behavioral test specific to the ICH-induced brain injury. This is different from the Parkinson's disease model that can be assessed with a 6-OHDA-induced behavior test stressed by apomorphine. Based on this fact, we enumerated and discussed the behavioral tests that have been used in preclinical ICH research to provide a clear guide for researchers. These behavioral tests include a full evaluation of pain, motor, cognitive, and emotional dysfunction. The rationale, setup, duration, baseline, procedures, as well as pros and cons of each assessment, are also discussed. One point to note is that the protocol and the baseline used in different laboratories may vary even with the same functional behavioral assessment.

There is a gap between preclinical and clinical research of post-ICH depression. Koivunen et al. reported that about one out of four ICH survivors suffers from long-term depression (168). Because of the high incidence of post-ICH depression, elucidating its pathomechanism and identifying the therapeutic strategies become hot areas of current stroke/ICH research. The application of emotion-related behavioral tests in preclinical ICH research will help with the screening of potential therapeutics for treating post-ICH depression. Through PubMed research, however, we identified only five research papers in which the depression-like behaviors were studied in rodents with ICH (56, 57, 75, 88, 169). Based on the fact that we know very little about post-ICH depression-like behaviors in rodents, more research into this new area is strongly recommended.

Because different brain regions control specific brain functions, the location of brain hematoma determines the type of dysfunction that results. The selection of the behavioral tests for ICH research should keep this in mind. For a striatal ICH model, locomotor function tests can be selected. For a cortical ICH model, the cognitive and emotional tests should be selected, and for a thalamic ICH model with restricted damage to the lateral posterior nucleus, the sensation, cognitive, and emotion-like tests can be selected. Of course, the selection of the specific behavioral test should consider the research objectives, the experimental conditions, and the available lab resources (64).

Several methodologic issues may have hampered the clinical translation of preclinical findings. To provide a feasible and precise assessment of drug efficacy and to elucidate the underly cellular and molecular mechanisms of action, researchers should select the appropriate behavioral tests associated with location-specific ICH-induced brain cell injury and relevant network dysfunction. Additionally, many variables in rodent behavioral tests including age, sex, the specific strain of the animals, and comorbidies such as diabetics and hypertension can all influence the animal's behavioral test performance. For instance, age-related decline in learning, memory, and sensorimotor functions are well-established observations (170–173). In this regard, careful characterization of the baseline behavior should be established to rule out the fundamental differences in test performance, especially if global knockout mice are included. Finally, the blinding strategy should always be followed to reduce the Pygmalion effect or the observer bias, which requires blind allocation of the experimental groups and the blind assessment of the outcome measures (174).

Although histology, cellular and molecular biology, genetics, and electrophysiology are key tools for understanding mechanisms of action of novel therapeutic strategies, behavior represents the functional outcome of ICH and should be used for the final preclinical evaluation. Good lab practice with careful selection and execution of existing behavioral tests as we discussed above may improve the outcome of future translational research.

## Author Contributions

XC and JianW: conceptualization. XS and HB: writing-original draft preparation. JiarW, LH, and JianW review, edit, and critical revision. All authors literature search, review, commentary, and final approval of the manuscript.

## Conflict of Interest

The authors declare that the research was conducted in the absence of any commercial or financial relationships that could be construed as a potential conflict of interest.

## Abbreviations

ART: Adhesive removal test
BW: Beam walking
EPM: Elevated plus maze
FPT: Forelimb placing test
FST: Forced swim test
GWT: Grid walk test
HLT: Horizontal ladder test
ICH: Intracerebral hemorrhage
MWM: Morris water maze
mNSS: Modified neurological severity score
NDS: Neurological deficit scale
NORT: Novel object recognition test
OFT: Open field test
PWL: Paw withdraw latency
RPM: Revolutions per minute
SPT: Sucrose preference test
TST: Tail suspension test
WHT: Wire hanging test.
